# Therapeutic Efficacy of Combining PEGylated Liposomal Doxorubicin and Radiofrequency (RF) Ablation: Comparison between Slow-Drug-Releasing, Non-Thermosensitive and Fast-Drug-Releasing, Thermosensitive Nano-Liposomes

**DOI:** 10.1371/journal.pone.0092555

**Published:** 2014-05-01

**Authors:** Alexander V. Andriyanov, Erez Koren, Yechezkel Barenholz, S. Nahum Goldberg

**Affiliations:** 1 Laboratory of Membrane and Liposome Research, Department of Biochemistry, Institute for Medical Research Israel-Canada, Hebrew University-Hadassah Medical School, Jerusalem, Israel; 2 Radiology Department, Hadassah Hebrew University Medical Center, Ein Kerem, Jerusalem, Israel and Beth Israel Deaconess Medical Center, Boston, Massachusetts, United States of America; Aristotle University of Thessaloniki, Greece

## Abstract

**Aims:**

To determine how the accumulation of drug in mice bearing an extra-hepatic tumor and its therapeutic efficacy are affected by the type of PEGylated liposomal doxorubicin used, treatment modality, and rate of drug release from the liposomes, when combined with radiofrequency (RF) ablation.

**Materials and Methods:**

Two nano-drugs, both long-circulating PEGylated doxorubicin liposomes, were formulated: (1) PEGylated doxorubicin in thermosensitive liposomes (PLDTS), having a burst-type fast drug release above the liposomes’ solid ordered to liquid disordered phase transition (at 42°C), and (2) non-thermosensitive PEGylated doxorubicin liposomes (PLDs), having a slow and continuous drug release. Both were administered intravenously at 8 mg/kg doxorubicin dose to tumor-bearing mice. Animals were divided into 6 groups: no treatment, PLD, RF, RF+PLD, PLDTS, and PLDTS+RF, for intra-tumor doxorubicin deposition at 1, 24, and 72 h post-injection (in total 41, mice), and 31 mice were used for randomized survival studies.

**Results:**

Non-thermosensitive PLD combined with RF had the least tumor growth and the best end-point survival, better than PLDTS+RF (p<0.005) or all individual therapies (p<0.001). Although at 1 h post-treatment the greatest amount of intra-tumoral doxorubicin was seen following PLDTS+RF (p<0.05), by 24 and 72 h the greatest doxorubicin amount was seen for PLD+RF (p<0.05); in this group the tumor also has the longest exposure to doxorubicin.

**Conclusion:**

Optimizing therapeutic efficacy of PLD requires a better understanding of the relationship between the effect of RF on tumor microenvironment and liposome drug release profile. If drug release is too fast, the benefit of changing the microenvironment by RF on tumor drug localization and therapeutic efficacy may be much smaller than for PLDs having slow and temperature-independent drug release. Thus the much longer circulation time of doxorubicin from PLD than from PLDTS may be beneficial in many therapeutic instances, especially in extra-hepatic tumors.

## Introduction

A main impediment of current anticancer chemotherapy is low tumor selectivity and the resultant generation of undesirable side effects [1]. The use of pharmaceutical nanoparticle carriers such as liposomes has been proposed as an effective approach to overcome these obstacles [2]. Nonspecific liposomal targeting is based on the pathophysiological phenomenon characterized as the enhanced permeability and retention (EPR) effect [3]. This occurs primarily in two main pathological states: inflammation and cancer, in which particles of approximately 100 nm or less preferentially accumulate in the diseased tissues. Benefits for liposome-delivered treatment of inflammation [4], [5] and cancer treatment [6], [7] have been reported *in vivo*. For nano-drugs to benefit from the EPR effect, the liposomes are required to evade the immune system. This is achieved by including a lipopolymer such as PEG-DSPE in the liposome membrane [8]. However, a selective accumulation at the tumor site by itself is not sufficient to achieve therapeutic efficacy. There is an obligatory need for a sufficient spontaneous drug release from the liposomes *in situ*.

Doxil is the first FDA-approved (and still extensively used clinically) liposomal nano-drug. The active pharmaceutical ingredient (API), doxorubicin, is remotely-loaded into PEGylated liposomes [8]. For Doxil, drug release at the tumor is assumed to be higher than in plasma in order to achieve the observed therapeutic efficacy. In the case of remotely-loaded Doxil, this drug release is also demonstrated by the presence of doxorubicin metabolites in the tumor tissue [8]. This is not the case for the passively-loaded “Stealth” cisplatin liposomes, which although they benefit from the EPR effect, unlike Doxil, do not show sufficient therapeutic efficacy *in vivo* due to insufficient spontaneous drug release at the tumor site [9], [10]. Although the mechanism of drug release in tumors from liposomes is not fully known, it is clear that it is dependent on three main factors: the mechanism of drug loading (remote versus passive), liposome membrane composition, and the tumor microenvironment [2], [8], [11].

Many studies have shown that external energy sources combined with appropriate lipid compositions resulted in improving controlled drug release at the tumor site, followed by improved therapeutic efficacy. Examples of external energy sources include heat, radiofrequency, ultrasound, and light [12]–[14]. Lipid composition of the liposome membrane is an important parameter which, in combination with energy use, controls the desired drug-release profile [5], [15], [16]. Liposomal membrane lipid composition influences drug release rate as a response to exposure to specific energy sources. For example, light-induced photochemical activation of content release from liposomes was previously designed to employ destabilization of membrane lipids by isomerization (azobenzene, retinoyl phospholipids, spiropyran, stilbene); cleavage (NVOC-DOPE, *o*-nitrobenzyl, coumarin);s or by polymerization (bis-sorb PC, diacetylene PC) of its components [17]. Furthermore, the use of *lyso*-phospholipids when combined with the lack of cholesterol for the construction of temperature-sensitive liposomes is a well-established strategy to achieve a fast burst release, as was demonstrated with ThermoDox [18].

Doxil membrane lipid composition, being based on the combination of high-Tm (53°C) hydrogenated soy phosphatidylcholine as the “liposome-forming lipid” [19] and a high mole % of cholesterol causes the liposome membrane to be in a liquid ordered (LO) phase, lacking the solid ordered (SO) to liquid disordered (LD) phase transition. This phase transition is typical of most phospholipids when cholesterol is missing from the lipid bilayer [15], [20], [21]. A lipid bilayer such as that of the non-thermosensitive liposomes, PLD, is expected to demonstrate a very slow, almost negligible zero-order release rate at 37°C *in vitro* in buffer and plasma, 5% after 30 h of incubation, as was shown previously [22], and also a slow release rate *in vivo* was shown by us (unpublished data). These liposomes are referred to as temperature insensitive liposomes. On the other hand, liposomal formulations that lack cholesterol exhibit a SO to LD phase transition at Tm’s, which depend on the exact lipid composition. Such liposomes are referred to as temperature-sensitive liposomes (PLDTS), and they burst-release their intra-liposome aqueous phase content upon passing through the phase transition [23]. One such PEGylated liposomal doxorubicin PLDTS formulation, referred to as ThermoDox, was developed by Needham and co-workers [24], [25]. This formulation showed promise in pre-clinical studies; However, recently it failed in a Phase III clinical trial for the treatment of liver cancer [26], [27]. This PLDTS lipid composition was designed to have its phase transition at 41.5°C, and once these liposomes are exposed to 42°C or higher they demonstrate a fast burst-type release of almost all loaded doxorubicin in less than 5 min. [28]–[31].

Given the availability of two very different liposomal formulations for the delivery of drugs such as doxorubicin, we can use them to optimize the therapy achieved by combining the liposomal drug delivery with RF. For this we have to answer two very critical questions:

1. What is the optimal drug release rate at the tumor tissue?
2. How stable should drug-loaded liposomes be in plasma?

It is also important to note that the answers to these queries may also depend on the mechanism of action of the anticancer drug [32], [33]. It may involve “phase-specific drugs”, which are most active against cells in a specific phase of the cell cycle (e.g. vincristine, which is specific to the mitosis phase) or for cell-cycle-specific drugs. The latter are effective while cells are actively in cycle, but do not depend on the cell’s being in a particular phase (doxorubicin belongs to this group). The third group is cell-cycle non-specific drugs (e.g. alkylating agents). The lack of answers to these two questions is often a barrier to achieving effective therapy.

PLDTS and Doxil (PLD) are two different approaches to deliver doxorubicin to ablated tissues, and represent two extreme situations. The PLDTS are long-circulating liposomes that demonstrate a solid ordered to liquid disordered phase transition (Tm of 41.5°C) and therefore have a heat-sensitive delivery of high doses of rapidly released drugs [34], whereas the second preparation (Doxil) are long-circulating liposomes, which, due to the high level of cholesterol, lack such a phase transition and, due to their high content of HSPC (Tm of 53°C), show a very slow release profile [5], [8], [35]. Thus, these two strategies offer two types of release kinetics that may lead to quite different strategies of doxorubicin delivery and of treatment clinical outcome. Both nano-drugs have been reported as exhibiting synergistic effects when combined with radiofrequency (RF) ablation [29], [36]–[38], gaining increasing clinical adoption with minimally invasive image-guided tumor therapy of focal hepatic, lung, renal, and bone tumors [39]–[41]. Increases in intra-tumoral drug deposition of 4–6 fold have been reported for both approaches [29], [36]–[38]. Since direct comparison of the two approaches has to our knowledge not yet been performed, we decided to compare them side-by-side using the same appropriate animal model. Specifically, in our current study, nude mice with human medulloblastoma [42] were treated with RF and two PEGylated liposomal doxorubicin preparations, as this chemotherapeutic is reported as a potent and active anticancer element for this type of cancer [43], [44].

## Materials and Methods

### Lipids and Doxorubicin

1,2-dipalmitoyl-*sn*-glycero-3-phosphatidylcholine (DPPC), 1-stearoyl-2-hydroxy-*sn*-glycero-3-phosphatidylcholine (MSPC) and N-carbamyl-poly-(ethylene glycol methyl ether)-1,2-distearoyl-*sn*-glycero-3-phosphoethanolamine sodium salt (PEG_2k_-DSPE) were obtained from Lipoid (Ludwigshafen, Germany). Doxorubicin hydrochloride (Dox) was obtained from Pharmachemie B.V. (Harlem, The Netherlands).

Doxil-like DOX-NP, PEGylated nano-liposomes remote-loaded with doxorubicin (PLD) by transmembrane ammonium sulfate gradient, was obtained from Avanti Polar Lipids (for details see: http://avantilipids.com/index.php?option= *com_content&view = article&id = 2331&Itemid = 607*) catalog #300102. DOX-NP (PLD) and PLDTS were characterized by us as described in Table 1 using the methodologies as described in [45] and reviewed in [8].

**Table 1:** Liposome characterization.

Nano-drug name	Lipid composition	Mole lipid ratio	Size (Z-average, nm)	PDI	ζ-potential(mV)	Tm (°C)
**PLD**	HSPC; Cholesterol; PEG-DSPE	57/38/5	84.4	0.05	−2.7±0.2	No phase transition*
**PLDTS**	DPPC; MSPC; PEG-DSPE	86/10/4	97.5	0.1	−1.7±0.2	42

*No phase transition is explained by the high mole % of cholesterol.

### Liposomal Preparation

PLDTS (PEGylated thermosensitive nano-liposomes remote-loaded with doxorubicin) were prepared according to previously reported methods [24], [25], [46]. Briefly, PLDTS were fabricated by lipid film hydration, followed by down-sizing using extrusion. Doxorubicin was remote loaded by transmembrane pH gradient using sodium citrate buffer as intra-liposome low-pH medium. Unloaded drug was removed by the cation exchange resin Dowex 50WX-4 (Sigma-Aldrich, St. Louis, MO, USA) or cation exchange column Stata-X-C Polymeric Strong Cation 200 mg/3 ml (Phenomenex, Torrence, CA, USA) [47]. The final amounts of intraliposomal doxorubicin were determined by using intensity of absorption at 480 nm using Synergy 4 Multi-Mode Microplate Reader (Biotek Instruments, Winooski, VT, USA), as previously described by Amselem et al. [48].

### Liposomal Characterization

Liposomes were characterized for their zeta-potential and size distribution by Malvern’s Zetasizer Nano ZS instrument (Worcestershire, UK), as described by Garbuzenko et al. [49]. Phospholipid concentration was determined using a modified Bartlett procedure [45], [50]. Differential scanning calorimetry (DSC) analysis was performed as previously described by Biltonen and Lichtenberg [51] using a MicroCal VP-Capillary DSC system (GE Healthcare, Uppsala, Sweden).

### Cryo-transmission Electron Microscopy (Cryo-TEM)

Cryo-TEM was used to confirm liposome size distribution measured by dynamic light scattering (DLS) and to characterize the detailed structure of the PLDTS before and after heat activation, as well as the physical state of the encapsulated drugs, as previously described [13].

### Drug Administration

The total amount of doxorubicin injected into the tail vein of each mouse in all groups, except control and RF only, was 8 mg/kg (up to 200 µL per mouse weighing about 25 g) [38], [41].

### RF Application

Conventional monopolar RF was applied by using a 500-kHz RFA generator (Model 3E; Radionics, Burlington, MA, USA). To complete the RF circuit, the animal was placed on a standardized metallic grounding pad (Radionics). Contact was assured by shaving the animal’s fur and liberally applying electrolytic contact gel. Initially, the 1-cm tip of a 21-gauge electrically insulated electrode (SMK electrode; Radionics) was located at the midpoint of the tumor. For all studies, duration of RF was 3 min, with the generator output titrated to maintain a designated tip temperature (70±2°C). This standardized method of RF application and the parameters chosen have been demonstrated previously to provide specific, constant dimensions of ablation with reproducible coagulation volumes, thereby enabling straightforward comparison of the effects of adjuvant drug administration [52].

Our protocols for administering liposomal doxorubicin in relation to the RF were optimized to maximum effect of either RF tissue changes (Doxil-PLD) [38], [53] or the equivalent of clinical recommendations for Phase III clinical trials combining thermally sensitive liposomes and RF [26]. These well-established treatment protocols are described in Schemes 1,2 (See supplementary data). For PLD treatment, drug was injected IV 15 min post-RF treatment to permit, on the one hand, induced changes in the tumor microenvironment that await the PLD, while at the same time allowing the tumor to be cooled and returned to baseline temperature [52], [54]. For PLDTS treatment, the opposite order was performed. PLDTS was administrated IV, and 15 minutes later the tumor was exposed to the RF, as a similar protocol was used previously by others including that for ThermoDox [26].

### *In vivo* Animal Model

Approval by the Institutional Animal Care and Use Committee of the Hebrew University (#MD-07-10404-5) was obtained before the initiation of these studies. The human medulloblastoma cell line (Daoy) was purchased from American Type Culture Collection (Manassas, VA, USA). Approximately 4 million Daoy cells were inoculated s.c. in the back of 4–5 week old NUDE-Hsd:Athymic mice (Harlan Laboratories, Jerusalem, Israel) [7]. For the bio-distribution study, and in order enable direct pair-wise comparison of the specific RF effect on the same mouse, two subcutaneous tumors were established by injecting 4 million Daoy cells in both the lower left and right flanks of each mouse. Four to six weeks after cell injection, tumors grew to the desired size (13±2 mm diameter for quantitation studies, and 11±2 mm for the survival study). For all experiments and procedures, anesthesia was induced with intraperitoneal injection of a mixture of ketamine (50 mg/kg, Ketaset; Fort Dodge Animal Health, Fort Dodge, Iowa, USA) and xylazine (5 mg/kg, Sedaxylan; Eurovet Animal Health B.V., Bladel, The Netherlands). Animals were sacrificed with an overdose of double the amounts of these drugs.

### Pharmacokinetics and Biodistribution

An initial group of 41 mice was randomized to six groups and sacrificed at 1, 24 and 72 h after the liposome intravenous administration (three time-points for each liposomal formulation, PLDTS and PLD). As mentioned above, in order to enable direct pair-wise comparison of the specific RF effect on the same mouse, RF was applied to all mice in one tumor only. An additional group of 8 mice (4 for each drug) was used for a 6-hour study. Recovered doxorubicin was evaluated in the most relevant tissues, including plasma, tumor (with or without RF treatment), and liver of all animals. Organs were harvested at the defined sacrifice times with samples stored at −80°C until analysis. The organs were homogenized in acidic isopropanol as described by Gabizon et al. [55]. Measurements were made by determining the fluorescence emission intensity at emission wavelength filter of 600±20 nm and excitation wavelength of 485 nm using Synergy 4 Multi-Mode Microplate Reader from Biotek Instruments (Winooski, VT, USA). The ability to distinguish between free and liposomal doxorubicin stems from the fact that the free doxorubicin at the concentration present in the plasma shows a lack of fluorescence quenching and demonstrates a linear increase of fluorescence with increasing doxorubicin concentration. On the other hand, the fluorescence of the Doxil liposome (encapsulated doxorubicin) is fully quenched [46]. When free and liposomal doxorubicin are mixed, only the fluorescence of free doxorubicin is expressed. Therefore, to determine plasma free doxorubicin concentrations for both formulations, we diluted 10 µL of plasma in 90 µL of physiological saline. For determination of total (free plus liposomal) doxorubicin, we dissolved the liposomal membrane by diluting 10 µL of plasma in 90 µL of acidic isopropanol [55], thereby releasing all liposome doxorubicin, as this large dilution from the very small trapped volume of the nano-liposomes to 100 µL causes a complete fluorescence dequenching allowing for the accurate determination of total (liposomal plus free) plasma doxorubicin.

The liposome-encapsulated doxorubicin was calculated by subtraction of free doxorubicin from total doxorubicin. For precise definition of tissues’ doxorubicin concentration (calculated following tissue weight measurements) presented as % of injected dose (%ID), we created a doxorubicin calibration curve and calculated the fraction of doxorubicin uptake into the various tissues based upon the following formula:
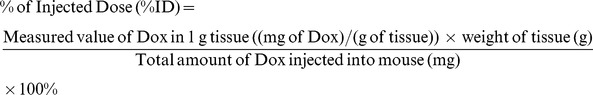



### Therapeutic Efficacy

On day zero, 31 mice with tumor size of 12×9±1 mm were randomized to six groups and treated as specified:

1. Mono therapy – injection of PLDTS
2. Mono therapy – injection of PLD
3. RF ablation alone
4. Combined therapy: PLDTS injection 15 minute before RF application [26]
5. Combined therapy: PLD injection 15 minute after RF treatment [52], [54], [56]
6. Control (non-treated mice)

Two primary endpoints were determined: growth rate and time to reach surrogate endpoint survival (burden of tumor more than 1000 mg). Tumor weights were calculated according to the equation:
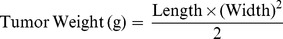



Direct caliper measurements were used for determination of tumor size [7], [57], [58].

Tumor size, survival, and body weight were monitored 2–3 times per week.

### Statistical analysis

Additional post-hoc analysis was performed with paired, two-tailed Student’s *t*-test, by using Prism 4 software (GraphPad Software, San Diego, CA, USA). A p-value of less than 0.05 was considered significant. The statistical significance between different treatment groups was determined using the 1-way ANOVA test for tumor volume and the log-rank (Mantel-Cox) test for comparison of Kaplan-Meir curves [59]. For determination of group diversity we used standard error values [60].

## Results

### Characterization of Liposome-loaded Drugs

Table 1 shows the PLD and PLDTS formulation parameters. The mean diameter of PLDTS was 97.5 nm with a polydispersity index (PDI) of 0.1. PLD showed mean a size of 84.4 nm with a PDI of 0.05. These results are similar to previously published data [8], [49].

**Figure 1:**
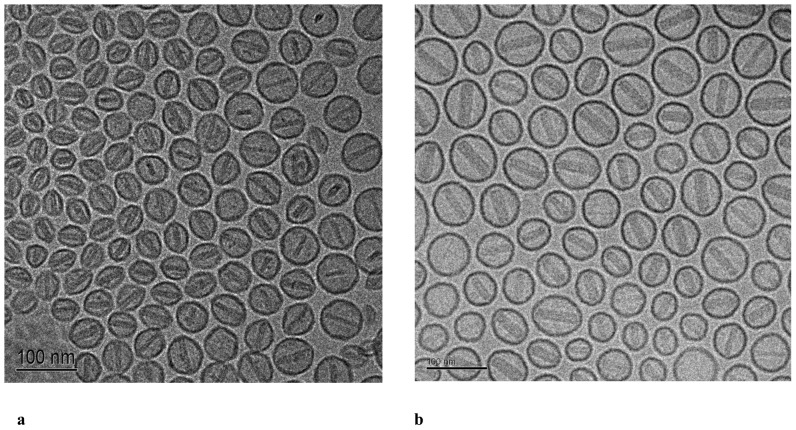
Cryo-TEM micrographs of PLDTS (a) and PLD (b) liposomal formulations at 25°C. Clearly seen are the ellipsoid liposomes containing the loaded doxorubicin, similar to what was previously observed for doxorubicin-loaded liposomes (Doxil/Myocet) [8], [23], [62].

Remote-loading efficiency of doxorubicin using ammonium sulfate gradient was ∼95% for PLD and ∼80.5% for PLDTS, in agreement with prior reports [28], [61]. Additionally, results of cryo-TEM show PLD and PLDTS remotely-loaded with doxorubicin (Fig. 1) with a similar liposomal doxorubicin “coffee bean shape” as previously demonstrated for Doxil [8], and for pH-gradient doxorubicin-loaded liposomes such as Myocet [62].

### Doxorubicin Pharmacokinetics and Biodistribution

The objective of the current study was to determine the thermogenic therapeutic effect of a PLD+RF combination therapy and to elucidate its mechanism of action (MoA). We compared two well-established tumor cancer treatment modalities: one, based on PLD, which has been previously shown to have a temperature-independent, slow zero-order release kinetics of doxorubicin [8] and the second, based on PLDTS, a temperature-sensitive and -dependent liposomal drug carrier, which shows fast burst-type release with almost complete release at temperatures slightly above the liposome membrane Tm of 41.5°C from solid ordered to liquid disordered, as described elsewhere [18] and confirmed by us using DSC for this study (data not shown). Our doxorubicin-release *in vitro* kinetics studies for both PLD and PLDTS liposomes were equivalent to those reported by others [34], confirming the relative equivalence of our preparations to theirs. Specifically, exposure of PLD in buffered saline to 4°C, 25°C, 37°C, and 42°C showed minimal release (i.e. less than 5% of the drug encapsulated) even after 48 h of incubation in a relatively temperature-independent manner. However, exposure of PLDTS formulation was highly temperature dependent and showed a burst release (81% over a 3 min exposure) at 42°C, above the liposome solid to liquid ordered phase transition temperature, as reported previously by others [25], [34] and complete drug release at 30 min exposure. The three lower temperatures tested (4°C, 25°C, and 37°C) showed only 0.3–0.5% drug release from PLDTS after three minutes, while after 30 min exposure the PLDTS showed a temperature-dependent release of 1.2%, 4.1%, and 9.7% of encapsulated doxorubicin at 4°C, 25°C and 37°C, respectively. This is related to the absence of cholesterol in the PLDTS and having DPPC as its liposome-forming lipid. One hour post-treatment initiation (See Table 2), both PLD and PLDTS modalities show a similar total doxorubicin fraction in evaluated tissues (with total recovery of ∼90%ID), which differ in their distributions between plasma and liver doxorubicin ratios ([free Dox+liposomal Dox]/[liver Dox]) (9.15 and 1.44 for PLD and PLDTS, respectively). In addition, percent of free Dox relative to total Dox plasma levels, as measured one hour post-treatment initiation, showed 13% and 5.0% of ID for PLDTS and PLD, respectively ([plasma free Dox]/[total plasma Dox]x100%). While one hour post-treatment initiation, plasma PLD doxorubicin was 76%ID, while for PLDTS doxorubicin it was only 42%ID, suggesting much faster clearance of PLDTS doxorubicin from the circulation already at 1 h post-treatment initiation.

**Table 2:** Biodistribution of doxorubicin following PLD and PLDTS administration.

		% of total Injected doxorubicin/organ					
Nano-drugname	Time (h)	Tumor+RF	Tumor(no RF)	Liver	PlasmaFree Dox	PlasmaLiposomal Dox	Total Dox recoveredin evaluated tissues
**PLD**	1	1.1±0.18	0.3±0.1	8.8±0.8	4.2±1.3	76.3±4.7	90.8±7.1
**PLD**	24	2.5±0.5	0.3±0.2	9.2±0.8	8.2±1.0	47.9±5.1	68.2±7.6
**PLD**	72	4.6±1.0	0.8±0.2	7.3±1.5	0.7±0.1	6.1±1.2	19.6±4.1
**PLDTS**	1	6.4±1.9	1.1±0.3	33.8±1.6	6.3±1.1	42.1±1.9	89.6±6.9
**PLDTS**	24	1.2±0.3	0.2±0.1	10.7±1.4	0.2±0.06	0.5±0.1	12.8±2.0
**PLDTS**	72	0.6±0.1	0.03±0.02	5.0±0.6	0.02±0.01	0.2±0.1	5.8±1.0

**Figure 2:**
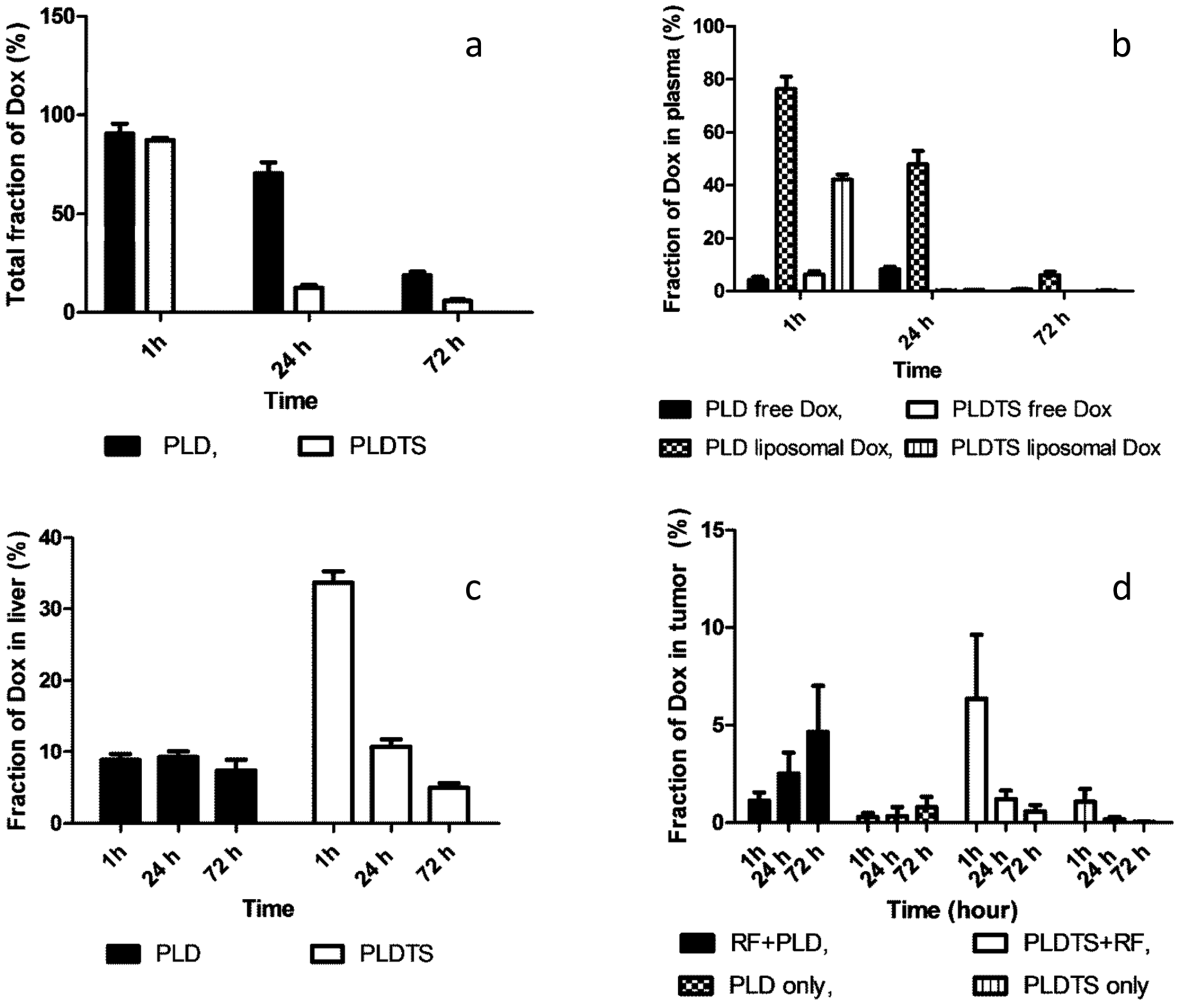
Doxorubicin biodistribution following PLD. (a) Total doxorubicin recovered in evaluated most relevant tissues; (b) Doxorubicin fraction in plasma, free and liposomal forms; (c) Doxorubicin fraction in liver; and (d) Fraction of intra-tumoral doxorubicin, with or without RF treatment. n = 4–7 per group. Statistically significant difference (p≤0.05).

Total doxorubicin levels in the most relevant tissues, plasma, liver, and tumors, were calculated for all time points of the experiment as the sum of all organs tested (Fig. 2a). It is clear that there was a reduction of doxorubicin levels over time in both PLD and PLDTS formulations. Nevertheless, the rate of decrease in total doxorubicin levels in the PLTDS-treated mice already at 24 h post-treatment (and very likely even earlier) is much larger, and this may shed light on the differences between the two formulations in therapeutic efficacy (to be discussed later). Our results show that from a similar starting point of ∼90% of ID, at 24 h post-treatment, the total doxorubicin recovered levels in evaluated tissues for PLDTS was reduced to 12.4% of ID, compared with 58% of ID for PLD (p≤0.01). Seventy-two hours post-treatment, the same trend is kept, although absolute values were lower for PLDTS (5.8% of ID for PLDTS and 19% of ID for PLD; p≤0.01).

Plasma levels analysis was conducted for both liposomal doxorubicin and free drug in plasma. At 1 h post-treatment, the fraction of free drug in plasma for both formulations lacked statistical significance (Fig. 2b). However a large (∼1.8 times higher) level of liposomal doxorubicin was observed for PLD. At 24 h, the difference between PLD and PLDTS was much more dramatic: for PLDTS the level of total doxorubicin was reduced drastically to less than 1% of ID, compared with more than 50% of ID for PLD-treated mice. Three days post-treatment, both nano-drugs show lack of free doxorubicin. The level of plasma liposomal doxorubicin 72 h post-treatment was still significant for PLD (6.1% of ID) but was at the limit of detection for the PLDTS-treated mice (0.2% of ID).

For the liver, an almost constant fraction of doxorubicin (7–9% of ID) was seen in mice treated with PLD (Fig. 2c) at all three tested time points (1, 24 and 72 h). However, with PLDTS, a rapid elevation at 1 h post-injection to ∼34% of ID, followed by a fast decline to 10.7% of ID at 24 h and 5% of ID at 72 h was observed, suggesting that a large fraction of doxorubicin was released from the PLDTS and reached the liver as a free drug.

The results of intra-tumoral drug concentrations (Fig. 2d) show different kinetics and maximal levels of doxorubicin for both treatment modalities, either for the nano-drugs alone or in combination with RF ablation. For combined PLDTS and RF ablation, maximum intra-tumoral drug was seen 1 h post-treatment initiation, while for PLD-RF treatment the highest accumulation was observed at the last time point checked, 72 h post-treatment initiation. It is possible that drug levels would be even higher at longer times post-administration [37], [38]. One hour post-injection, the PLDTS shows 6-fold higher intra-tumoral doxorubicin levels when compared to PLD (6.4% and 1.1% of ID, respectively). At 24 h post-injection, the doxorubicin tumor levels were reduced dramatically for PLDTS treatment (1.2% ID), while it doubled for PLD (2.5%ID). Of special interest are the findings that tumor accumulation for the PLD treatment kept increasing with time, reaching at 72 h the level of 4.6% of ID compared with very low levels of only 0.6%ID for PLDTS. Thus, reversed trends of increasing intra-tumoral drug for PLD and decreasing drug for PLDTS were found. The nano-drug with RF combination treatment was shown to be more efficacious than PLD or PLDTS treatments alone for both nano-drugs. However, since prolonged exposure of cancer cells to doxorubicin is clinically preferable and is a more efficient treatment approach, the PLD and RF ablation combination clearly implies a superior protocol (in respect to tumoral doxorubicin fraction) compared to the other treatment protocols evaluated in this study at 72 h post-treatment initiation and resulted in the highest doxorubicin fraction of %ID.

### End-point Survival Studies

**Figure 3:**
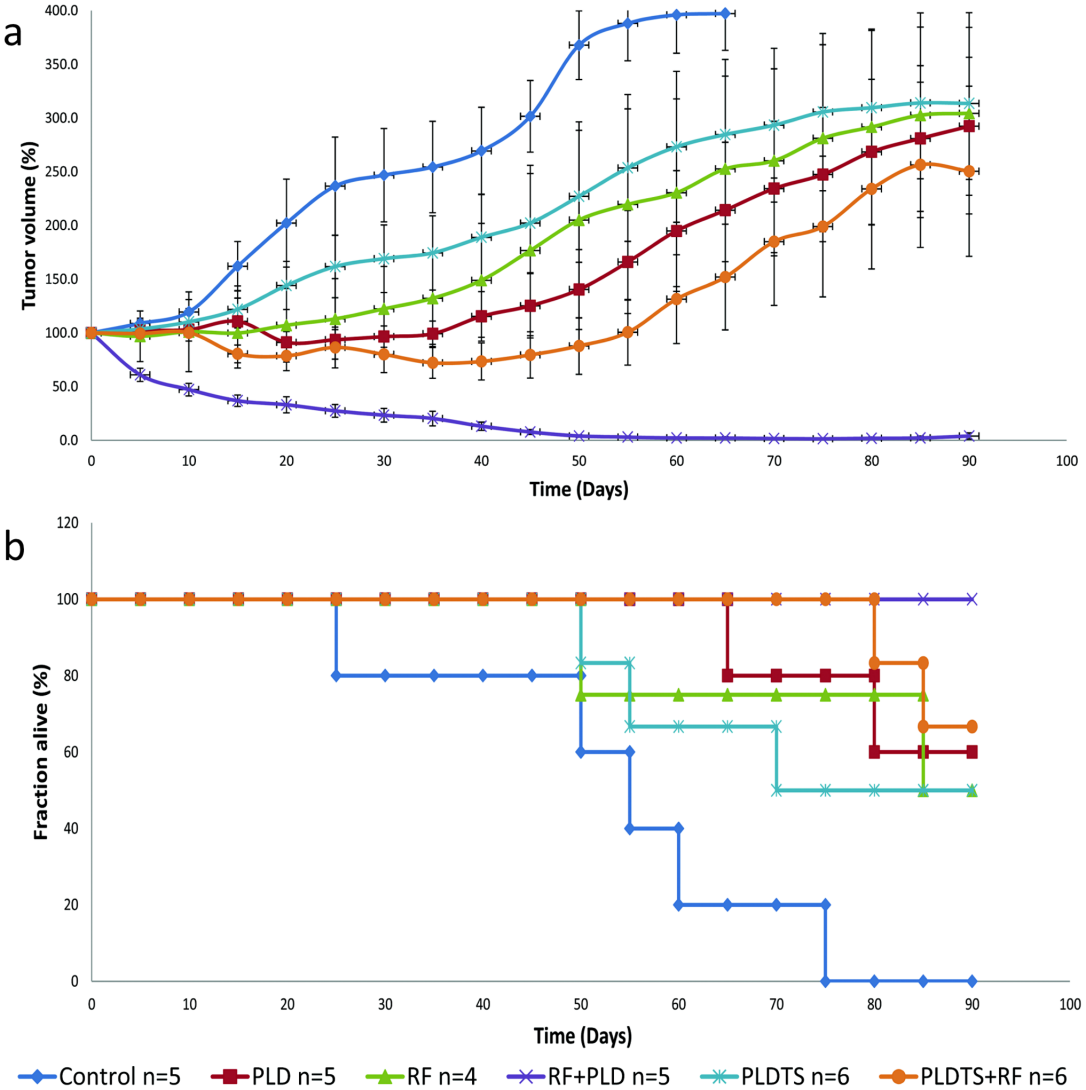
Mice tumor model therapeutic efficacy studies using PLD and PLDTS with and without RF ablation. (a) Effect of the various treatments on tumor volume during 90 days duration, and (b) survival of mice by using surrogate end point.

Comparison of the two therapeutic approaches was assessed via a survival study during 90 days follow-up. The analysis is based on using the “humane end point” of 4-fold tumor growth (as required by the animal ethics committee). Change in tumor volume was seen in mice of all groups (Fig. 3a). All single therapy groups (RF, PLD, and PLDTS) did not show statistically significant differences among them, and all displayed increased survival compared to the control group (p<0.0001) but lower survival compared to both combination therapy groups (PLDTS with RF [p<0.005] and PLD with RF [p<0.0001]).

Most importantly, the combination of RF with PLD was much superior to RF+PLDTS, with high statistical significance. Only the RF+PLD treatment showed significant long-term reduction of tumor volume and macroscopic disappearance of tumor. This is also the only group that showed 100% survival (by the humane end point) for at least 90 days compared to all other treatment protocols.

Kaplan-Meier survival curve results (Fig. 3b) demonstrate that all mice from the group treated with combined therapy of RF and PLD survived to the last day of experiment. By contrast, the last mouse from the control group was sacrificed at day 70. 50% of mice from the two single-therapy groups: RF and PLDTS were alive on the last day of experiment. 67% and 60% of mice treated with PLDTS plus RF and with PLD alone, respectively, survived to the end of the experiment. Thus, we observed a statistically significant differences between mice treated with the combination therapies (RF with PLD and RF with PLDTS) (p<0.05).

Almost all comparisons except RF vs. PLD, RF vs. PLDTS, and PLDTS+RF vs. PLD had statistically significant differences between them (p<0.05).

## Discussion

Tumor microenvironment can be altered following RF ablation, and therefore timing between the combined treatments is crucial for success. Goldberg et al. [63] treated several different liver malignancies, including 4 patients with hepatocellular carcinoma, and were able to attain 25–30% increases in coagulation volume by administering liposomal doxorubicin 24 h before RF application. Follow-up imaging studies demonstrated that this particular form of adjuvant therapy resulted in more complete tumor destruction because coagulation progressed over time to include residual tumor foci and patent intra-tumoral blood vessels. Thus, measured pathological changes and coagulation diameter over time can serve as an effective estimation tool to elucidate the described combined treatment. Accordingly, it was previously shown [52] in a rat breast cancer tumor model treated with combined RF and liposomal doxorubicin-loaded liposomes that for tumors treated with both liposomal doxorubicin and RF ablation therapy, the increase in observed coagulation diameter was progressive to 48 h post-RF ablation, with equivalent coagulation diameters observed when the liposomal doxorubicin was administered 3 days before to 24 h after RF application. It was also previously shown [38] that radiofrequency thermal ablation sharply increases intra-tumoral liposomal doxorubicin accumulation as well as tumor coagulation, with intra-tumoral doxorubicin accumulation increased to a maximum at 72 h with greater uptake in the RF-ablated tumors compared with controls or with liposomal doxorubicin alone at all time points in the R3230 mammary adenocarcinoma rat model. Additionally, RF enabled even greater uptake in tumor compared with liver. Further proof of liposomal doxorubicin altering the RF ablation-induced microenvironment has recently been published by our group [16], noting that RF ablation induces morphologic changes to vessels within the ablation zone lasting 12–24 hours after treatment, but that the addition of liposomal doxorubicin causes early vessel contraction and a reduction in peri-ablational microvascular patency, and continuing: “Such changes would likely need to be considered when determining optimal drug administration and imaging paradigms”.

Two different mechanisms can be suggested to explain Doxil’s doxorubicin internalization into tumor cells in vivo:

1. Uptake of intact Doxil liposomes by cells, followed by intracellular drug release;
2. Doxorubicin release in the tumor interstitial fluid, from where it is taken up by cells as a free drug.

The contribution of the intact Doxil uptake by tumor cells must be minimal, as intact cisplatin Stealth nano-liposomes, which have a similar lipid composition and size distribution as Doxil, did not show uptake of cisplatin by tumor cells and therefore showed lack of therapeutic efficacy (see [9]). Therefore, we are left with the second option of tumor cells’ uptake of drug that was released in the tumor interstitium. Factors leading to doxorubicin release from Doxil may include collapse or partial collapse of the ammonium sulfate gradient and/or the destabilization of Doxil liposomes by phospholipases that hydrolyze the liposome phospholipids (see review by Mouritsen and Jørgenson [64], thereby enabling faster doxorubicin release. However, there are two major objections to the latter phospholipase related drug release explanation. The first one is the fact that there is no drug release in vivo from Stealth cisplatin, which is identical in size and lipid composition to Doxil; the second is that the presence of cholesterol in the liposome membrane inhibits drastically phospholipase activity [64]. Therefore, we are left with the default, which suggests that the collapse of the ammonium sulfate gradient plays a more major role in doxorubicin release of Doxil in vivo. However, the latter assumption is as yet unproven and its proof requires further in-depth investigation. In the present communication, we compared two clinically-relevant methods of combining long-circulating PEGylated nano-liposomes encapsulating doxorubicin and RF ablation for the treatment of human medulloblastoma cancer using an extrahepatic tumor xenograft mouse model. To achieve this goal, we formulated PLD and PLDTS liposomes and we showed that they have similar properties to those previously reported [18] (see Table 1). Both nano-drugs used are PEGylated nano-liposomes, are of a similar size distribution and both include a similar mole fraction of 2000-Da PEG-DSPE, and therefore the liposomes in both cases are long-circulating sterically-stabilized. Structure analysis based on cryo-TEM shows that both formulations, when loaded with doxorubicin, show a similar ellipsoid “coffee-bean” structure, as previously described for PLD [8], [62]. The large and quantitive burst in drug release at or above Tm is a well-established phenomenon [15] and was confirmed in this study as well (data not shown). Such rapid release of drug from the liposomes is dramatically increased when *lyso-*palmitoyl phosphatidylcholine (LPC) is a liposome-membrane component [28]–[31], [65]. A complete and fast drug release from PLDTS above the solid ordered to liquid disordered phase transition temperature is contributed by the presence of LPC. These results are in agreement with previously reported heat-induced doxorubicin release of ∼90% from PLDTS in the first 5 min of exposure to 42°C [25], as was also confirmed by us.

PEGylated liposomes or other nano-drugs can passively accumulate in targets via the EPR effect [3]. Furthermore, when designing a “smart” drug delivery platform, specific characteristics of tumor microenvironment can further enhance the delivery or release of a variety of agents to a desired target, with extracellular release or intracellular distribution to specific organelles [66]. Tumor microenvironment-sensitive carriers were previously designed and demonstrated *in vitro* and *in vivo* changrs by manipulation of the carrier’s swelling behavior, network structure, permeability, or stability in response to tumor-environmental changes (e.g. pH, ionic strength), as well as following local exposure to elevated temperature by ultrasound or RF ablation [12], [13], [67], [68]. In relation to the drug carrier used, an absence of these factors can potentially lead to an unsatisfactory drug release profile and low drug levels at the desired site. The outcome might be a dysfunction of therapy, as noted for liposomal cisplatin, where poor anticancer therapy has been attributed to a lack of sufficient intra-tumoral drug release in spite of superior intra-tumoral accumulation of the nano-drugs [8], [10]. Earlier, we studied the use of RF ablation combined with intravenously administered PLD, and compared this with the use of RF ablation or doxorubicin alone [57]. These studies demonstrated that the RF+PLD combination facilitated and increased tissue coagulation and tumor interstitial drug accumulation in animal models [38]. However, the optimal drug release rate at the tumor site and its potential concomitant effect of altering the tumor microenvironment remained an open question whose answer may be highly beneficial for further improving the therapeutic efficacy of liposome-based nano-drugs.

Therefore, here we purposefully compared the two extremely different situations: (a) temperature-driven, fast burst-type release and (b) passive, temperature-insensitive, slow drug release. For both cases we compared the performance of the two systems with and without changing the tumor microenvironment using RF. Our PLD was constructed to be identical to Doxil, a non-thermosensitive liposome where membrane is in the liquid-ordered (LO) phase. Doxil was approved by the FDA in 1995 and has been in clinical use since [69]. Our second formulation, PLDTS, was constructed to be similar to ThermoDox, a formulation that recently has been studied in Phase III clinical trials [26]. It has a fast drug release above its 41.5°C Tm. Our injection protocol is designed in such a way that a significant local (at site of RF) drug release from the liposomes occurs upon exposure to RF heating. It also worth noting that for PLDTS, 1 h post-injection, after the exposure to RF, almost 90% of doxorubicin ID was still retained in the animal body, mainly in the plasma (48.4%ID total plasma concentration, 42.1%ID encapsulated and 6.3%ID free) and in the liver (∼34%ID) (Table 2). However, these values decay very fast with time (Table 2). This significantly poorer performance of PLDTS is related to the fast release of doxorubicin from the PLDTS even without exposure to RF, as is obvious from the very low drug retention in the total evaluated doxorubicin levels in plasma, liver, and tumors of PLDTS-treated mice (Table 2). Specifically, doxorubicin retention after administration of PLDTS is too low to enable drug-loaded liposome accumulation at the tumor site.

For PLD (which is not thermosensitive) we used a different established protocol. Here, the tumor was first exposed to RF to induce changes in tumor microenvironment, and 15 min post-irradiation (after the tumor cooled to baseline) PLD was injected. As described above, PK, BD, and efficacy of the two formulations differed to a large extent. Data analysis of biodistribution experiments shows that 1 h after injection, most of the drug measured in the plasma was retained in the liposome. This strongly suggests that at 15 min post-PLDTS injection, immediately before the time of tumor exposure to RF, the level of plasma PLDTS doxorubicin was significantly higher than 48.4% of ID (total plasma concentration) at 1 h and mostly as liposomal drug. This observation, however, raises an important question: why with RF ablation 6 h post-PLDTS injection do we not achieve similar levels of doxorubicin in the tumor or plasma as we observed for the PLD? We hypothesize that for this treatment protocol and this liposomal composition, liposomes might be “empty”, namely lacking a significant level of doxorubicin, which probably occurred due to the much faster release than from the PLD, which show very slow drug release in plasma.

For PLDTS, the short interval between the injection and the exposure to RF was a must, as we wanted to have enough drug-loaded liposomes remaining in the blood circulation. We demonstrated that when RF exposure is performed much later post-injection, there are insufficient drug-loaded liposomes in the circulation. It is suggested that this is due to fast doxorubicin release from the PLDTS, which at 37°C show ∼10% (zero order) release in the 30-min incubation. This explains the lack of doxorubicin in plasma 6 h after PLDTS administration (data not shown), leaving behind circulating “empty” PLDTS. Therefore, the desired efficacy is not achieved.

The difference in release rate is related to the PLDTS lacking cholesterol and also having DPPC (Tm at 41.5°C) as the liposome-forming lipid, while the PLD is based on the high-Tm (53°C) HSPC as the liposome-forming lipid and on a high mole % cholesterol, which abolishes the phase transition. Therefore, as expected, the PLD is much more release-resistant than the PLDTS and as the process of tumor nano-drug accumulation is a slow process, PLD has a large advantage over the fast-releasing PLDTS. It is important to note that although the role of cholesterol in this effect is dominant, the contribution of the PC used cannot be ignored. Long ago we showed that PLD based on DPPC as a liposome-forming lipid has poorer drug retention, when compared with PLD based on HSPC as the liposome-forming lipid. That is one of the main reasons why HSPC was the liposome-forming lipid of choice for Doxil [46].

As expected, determination of doxorubicin concentration in liver, plasma, and tumor demonstrates that PLDTS and PLDs result in two very different pharmacokinetics profiles. The relatively fast decrease of total doxorubicin fraction of PLDTS compared to PLD may be explained by zero-order kinetics in the case of PLD, compared to first or even second-order kinetics in the case of PLDTS. Thus, whereas the liver drug fraction after injection of PLD was constant from 1 to 72 h (about 10%), those after PLDTS injection displayed an exponential decay with drug concentrations significantly higher (33% of ID) at 1 h (p<0.0001) and only 5% by 72 h. This may be predicted by the significant decrease in plasma total doxorubicin levels 1 h post-PLDTS injection, from 48.4% total plasma concentration (while total body doxorubicin is ∼90%ID) to 0.7% total plasma concentration at 24 h (with total body doxorubicin of 12.8%ID). This profile of rapid clearance was also observed by Poon and Borys in clinical studies [29] and by Gasselhuber et al. in another small animal model [70]. Likewise, the presence of PLD liposomes in the blood stream for at least three days is similar to that noted previously [8], [70].

Key findings of our work include that there are substantial differences in drug profile dynamics observed in the most clinically relevant tissue assessed, the intra-tumoral doxorubicin fraction. These differences were magnified especially in combination with RF, as the application of heat fulfilled a different adjunctive role for each nano-drug. In the case of PLDTS, RF heating essentially acts as a trigger for drug release, and thus RF was applied in the presence of nano-drug in the body. Thus, although for both preparations, combined therapy leads to a significantly high doxorubicin fraction, for PLDTS the maximum of drug fraction in tumor was observed after the first hour. By contrast, in the case of PLD, RF was performed immediately prior to drug injection to specifically lead to the intensification of EPR effect due to change in tumor microenvironment. The concentration of the long-circulating drug, even after the RF-ablation heat effect was no longer present, was higher for PLD, but even for the PLDTS it was still significant and may result in better therapeutic efficacy if drug retention in plasma liposomes could be improved. Thus, the use of PLD benefits from RF’s inflammatory reaction without bursting the nano-carrier [41]. Accordingly, the intra-tumoral fraction of doxorubicin in the first hour was minimal and reached its maximum after three days or even longer.

The biodistribution study largely forecast the results of our therapeutic efficacy study. Based upon prior reports [37], it is not surprising that improved anticancer effects were obtained with combined therapy. Specifically, for the first 40 days, RF combined with either liposomal preparation resulted in negative tumor growth, whereas, by comparison, all single therapy groups, as well as control demonstrated positive tumor growth. Yet, over the next 50 days, differences between the two preparations in combination with RF began to become apparent, as PLDTS+RF likewise began to demonstrate tumor growth while RF+PLD did not show tumor growth. Indeed, no RF+PLD mouse demonstrated recurrence of tumor and as a result demonstrated 100% survival. However, for the other groups, end-point mortality was proportional to the rate of tumor growth, being markedly reduced in the combined therapy of the RF and PLDTS group and maximal in the control group.

We initially performed experiments to determine whether greatest synergy between RF and doxorubicin occurred with high doses of drug released immediately during the ablation process (facilitated by a thermally sensitive liposomal carrier) vs. a process of exposing the partially RF-treated tumor to a relatively long exposure of drug (using a long-circulating liposomal carrier). In a manner akin to Aesop’s famous fable of the tortoise and the hare, our results confirmed greater intra-tumoral drug concentrations at 72 h and greater survival of the Daoy cell-line-bearing mice for the PLD preparation. Furthermore, we noted that for extra-hepatic tumors in mice, the much faster release of doxorubicin from PLDTS, ironically, did not have the intended effect of greater long-term intra-tumoral drug concentrations, as the overwhelming majority of freed doxorubicin was found in the liver within the first hour of drug delivery and ablation. Indeed, overall, more drug stayed in the extra-hepatic tumor over a longer period of time when using PLD, and this is likely to have influenced the difference in survival noted. Thus, our results again underscore that it is not enough to deliver a drug to the target site, but that our goal must be modified to have long and sufficient exposure to released drug in order to have the desired local therapeutic target effects. Clearly, for some tumors and/or chemotherapeutics (such as for doxorubicin [71]), contact time is paramount as it may give more opportunities for a drug to interact favorably with its intended target. For drugs that are most efficacious in dividing or growing cells, a longer window of opportunity enables a larger portion of cells to be affected. By the same token, one can ask of the thermally-sensitive liposome approach: if we burst the liposomes, release the contents, and the active agent doesn’t stay *in situ* long enough to have optimal effect, of what benefit was using the liposome to increase the intra-tumoral concentration in the first place?

Our study also highlights ways to improve RF ablation therapy. One important item to note in our study is the fact that we used clinically relevant drugs and therefore translations to early clinical trials as a next step are certainly feasible [41]. The double stress caused by both RF and the drug selected showed anti-tumoral synergism, making this work more clinically relevant.

We understand the limitations of our present work. Most importantly, we acknowledge that we used only one animal model. Thus, it is quite possible that a strategy involving thermally sensitive liposomes may perform better in other models. There is an abundant literature advocating high first-pass concentration chemo for hepatocellular carcinoma that may support a PLDTS approach for hepatic cancer [72], [73].

Nevertheless, our study sets the stage for a call for study on a cancer-by-cancer basis in clinical trials. Further work on other nano-drugs and understanding their effect over time also needs to be performed, as well as the effects of other doses of RF energy and other thermal energy sources clinically used, such as microwave, ultrasound, and laser [12].

## Conclusions

Substantial variability in outcome can be seen when using different strategies for administering liposomal drugs in relation to RF ablation. Specifically, we report that a strategy based on using RF ablation with long-circulating liposomes resulted in longer intra-tumoral drug retention and end-point survival than a strategy based on a thermally-dependent burst of nanoparticles leading to overload of anticancer agent in the Daoy mouse tumor model. Thus, at least for some tumors, the timing and length of exposure to doxorubicin may be more important than shorter courses of high concentration chemotherapeutics. Our study stresses that the stability of the nano-drug, especially with respect to drug release in plasma at body temperature is critical for the nano-drug performance. Therefore, the evaluation of PLDTS formulations, with better liposomal drug retention *in vivo* than the PLDTS used here, is needed to better understand the potential of PLDTS as nano-drugs. The large impact of RF on the therapeutic efficacy of nano-drugs described here and before [26], [37]–[41], [52], [54], [74] encourages further research aimed at exploring optimal methods of combining ablation therapy and nano-drugs.

## Supporting Information

Data S1 **Experimental design, PK.**
(TIF)

Data S2 **Experimental design, Survival.**
(TIF)
