# Supplementary figures and images for: Therapeutic Efficacy of Combining PEGylated Liposomal Doxorubicin and Radiofrequency (RF) Ablation: Comparison between Slow-Drug-Releasing, Non-Thermosensitive and Fast-Drug-Releasing, Thermosensitive Nano-Liposomes
Source: PLoS One. 2014 May 1;9(5):e92555. doi: 10.1371/journal.pone.0092555 (PMC4006748; doi:10.1371/journal.pone.0092555)

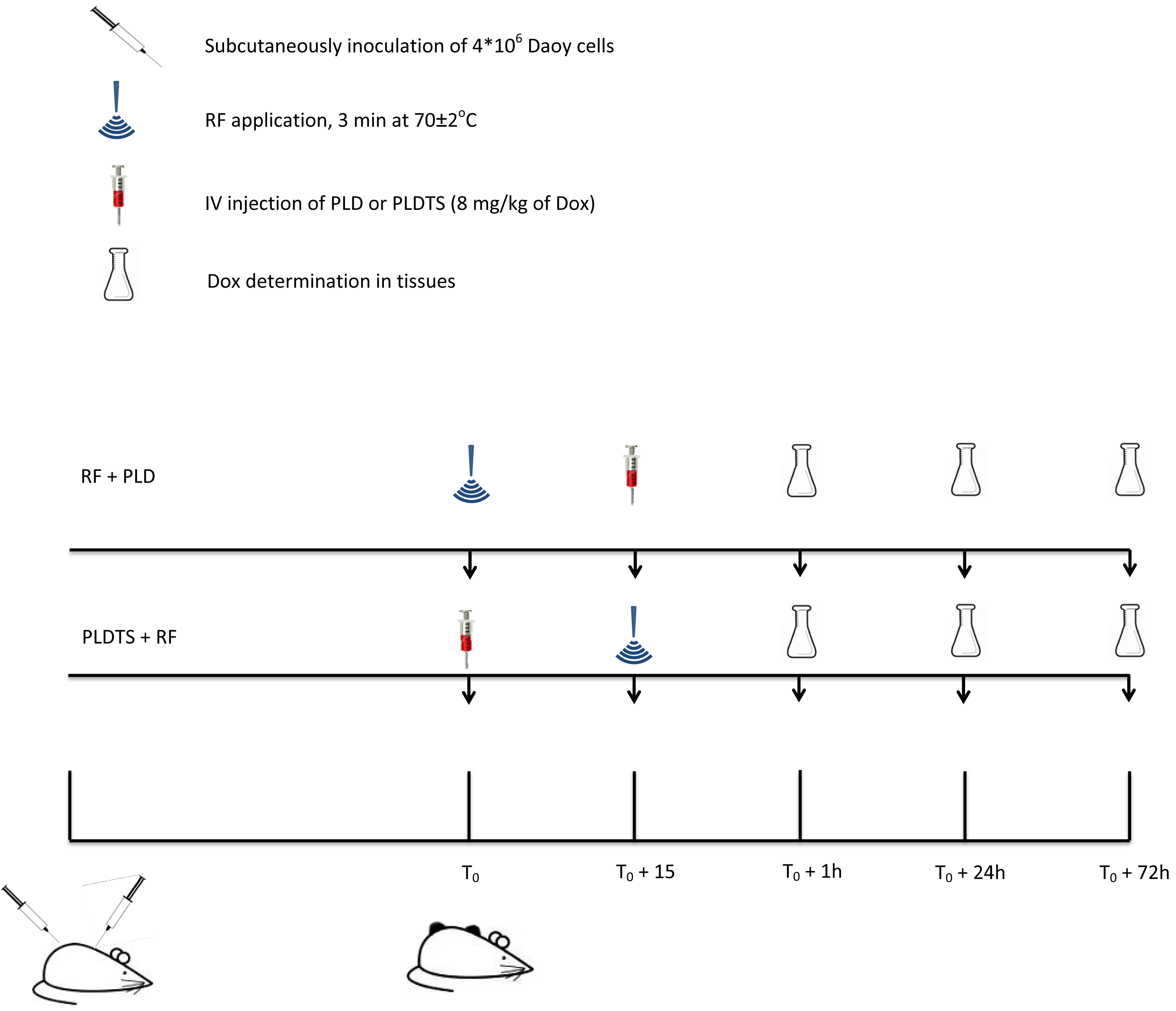

Supplement: Data S1 — Experimental design, PK. (TIF) [file pone.0092555.s001.tif]

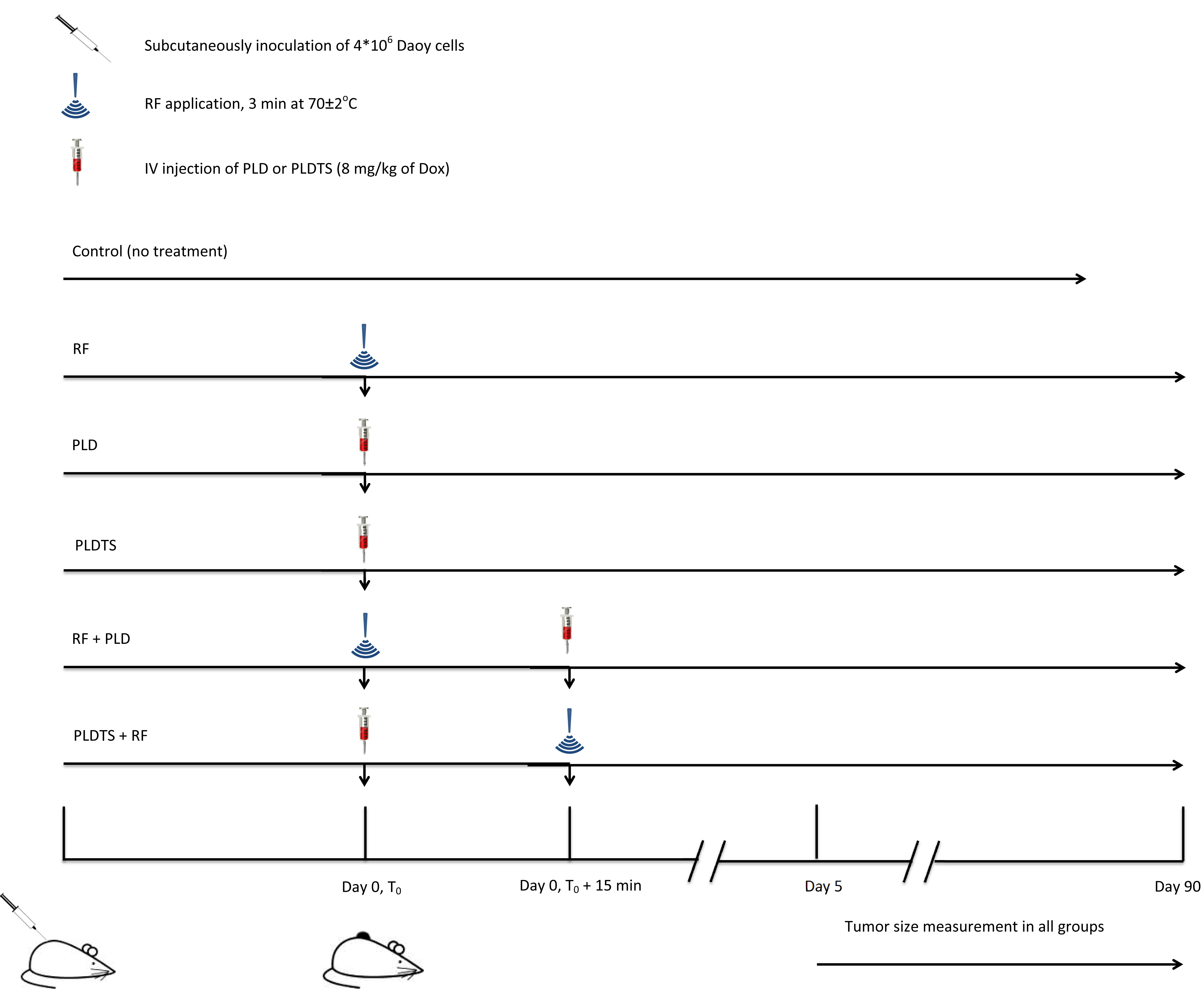

Supplement: Data S2 — Experimental design, Survival. (TIF) [file pone.0092555.s002.tif]
